# Real-world study of telitacicept in the treatment of IgA nephropathy

**DOI:** 10.3389/fimmu.2026.1810706

**Published:** 2026-05-29

**Authors:** Ling Hu, Meijuan Meng, Yihan Jiao, Jie Yang, Yun Fan, Xiaobin Liu, Bin Liu, Liang Wang

**Affiliations:** Department of Nephrology, The Affiliated Wuxi People’s Hospital of Nanjing Medical University, Wuxi People’s Hospital, Wuxi Medical Center, Nanjing Medical University, Wuxi, China

**Keywords:** 24-hour proteinuria, estimated glomerular filtration rate, IgA nephropathy, remission rate, telitacicept

## Abstract

**Purpose:**

To evaluate the efficacy and safety of telitacicept for the treatment of immunoglobulin A nephropathy (IgAN).

**Methods:**

This study enrolled patients with biopsy-confirmed IgAN who had 24-hour proteinuria exceeded 0.75 g/day and received telitacicept treatment for at least 3 months. Using propensity score matching, patients were matched in a 1:1:1 ratio with those who received only supportive or immunosuppressive (IS) therapy (glucocorticoid with or without mycophenolate mofetil). The primary outcomes were percentage changes in 24-hour proteinuria and estimated glomerular filtration rate (eGFR) during the 12-month follow-up period.

**Results:**

Each group included 24 patients. At 12 months, telitacicept reduced 24-hour proteinuria by 1.50 g/day (57.61%) from baseline, while the supportive treatment group had a reduction of 0.60 g/day (17.11%) and the IS treatment group had a reduction of 1.60 g/day (59.49%). The percentage change in 24-hour proteinuria in the telitacicept group was similar to that in the IS treatment group, and both groups were superior to the supportive treatment group. The telitacicept group exhibited a minor decline in eGFR of 2.1 mL/min/1.73 m^2^ (−4.23%), while the supportive treatment group and IS therapy group showed declines of 6.05 mL/min/1.73 m^2^ (−13.87%) and 2.65 mL/min/1.73 m^2^ (−5.65%), respectively. The incidence of adverse events was lower in the telitacicept group than that in the IS treatment group. Meanwhile, there was no statistical difference in the remission rates (including complete remission and partial remission) between the telitacicept and IS treatment groups, and both groups were superior to the supportive treatment group.

**Conclusion:**

Telitacicept can reduce 24-hour proteinuria in patients with IgAN and stabilize eGFR with good safety during follow-up. These findings suggest that telitacicept may represent a safer and effective therapeutic alternative to conventional IS regimens for reducing proteinuria and preserving renal function in patients with IgAN, particularly those at high risk of disease progression.

## 1 Introduction

Immunoglobulin A nephropathy (IgAN) is the most common primary glomerular disease in China and is characterized by mesangial deposition of immunoglobulin A (IgA), representing a leading cause of end-stage renal disease (ESRD). Approximately 20–40% of patients with IgAN progress to ESRD within 10–20 years after initial clinical manifestation (1).

The pathogenesis of IgAN is multifactorial and is largely driven by dysregulated immune activation. In IgAN, pathogenic IgA-containing immune complexes form and deposit in the kidneys, triggering inflammatory cascades that impair renal function and glomerular filtration. The widely accepted “four-hit” hypothesis proposes that mucosal immune stimulation induces B cell activation via B cell activation factor (BAFF) and a proliferation-inducing ligand (APRIL), leading to class switching toward IgA-producing plasma cells (2, 3). This process results in increased circulating levels of galactose-deficient IgA1 (Gd-IgA1). Subsequent autoantibody formation against Gd-IgA1 promotes the assembly of nephritogenic immune complexes that deposit in the glomeruli, inducing mesangial proliferation, cytokine release, and complement activation, ultimately causing inflammation and renal damage (4). Current treatments for IgAN primarily emphasize supportive management, including blood pressure control, reduction of proteinuria, modification of cardiovascular risk, and lifestyle interventions (5). However, these strategies fail to fully prevent disease progression, particularly in high-risk patients. The TESTING study (6) demonstrated that despite supportive care and glucocorticoid therapy, patients with IgAN remain at substantial risk of progression to kidney failure within 10 years. Accordingly, there is an urgent need for effective and safe targeted therapies that can delay the onset of ESRD in high-risk patients with IgAN.

Telitacicept is a fully human, dual-target transmembrane activator and calcium-modulating cyclophilin ligand interactor (TACI)-Fc fusion protein developed using recombinant DNA technology (7). By linking the extracellular domain of the B cell surface receptor TACI to the Fc region of IgG1, telitacicept simultaneously binds APRIL and BAFF, thereby inhibiting B cell maturation and proliferation. This mechanism reduces the secretion of Gd-IgA1 from mucosal plasma cells and may slow disease progression (8). We aimed to conduct a single-center retrospective study to evaluate the efficacy and safety of telitacicept and to provide clinical evidence supporting its use in IgAN.

## 2 Materials and methods

### 2.1 General information

A retrospective analysis was conducted in patients with IgAN treated with telitacicept at the Affiliated Wuxi People’s Hospital of Nanjing Medical University. The study was approved by the Ethical Review Board of the Affiliated Wuxi People’s Hospital of Nanjing Medical University (clinical trial registration number: KY24040).

We recruited and screened patients with IgAN treated between February 2022 and May 2025. The inclusion criteria were as follows: (1) diagnosis of IgAN via biopsy, (2) age between 18 and 65 years, (3) 24-hour proteinuria > 0.75 g/day after receiving at least 12 weeks of standard supportive therapy; (4) 12 months of follow-up data. The exclusion criteria for the three groups were as follows: (1) secondary IgAN, (2) comorbid autoimmune diseases, (3) history of active hepatitis or severe liver disease, (4) history of malignant tumors, (5) history of severe infections, and (6) incomplete or missing data.

We matched the telitacicept group with the supportive and IS treatment groups by propensity matching for age, sex, baseline 24-hour proteinuria, baseline creatinine, and baseline estimated glomerular filtration rate (eGFR). Detailed baseline data and matching results were shown in **Supplementary Tables 1**-**4**. All patients in the three groups received supportive treatment for at least 12 weeks. The telitacicept group received telitacicept treatment, without the combination of glucocorticoids or other IS therapy. Patients in the supportive treatment group received the maximum tolerated dose of renin-angiotensin-aldosterone system inhibitors (RAASi), with or without sodium–glucose transport protein 2 inhibitors (SGLT-2i). The IS treatment group received glucocorticoids with or without mycophenolate mofetil (MMF), with doses of 0.5 to 1 mg/kg/day for glucocorticoids and 1 to 2 g/day for MMF.

### 2.2 Data collection

General data were collected, including age, sex, body mass index, disease duration, blood pressure, comorbidities, concomitant medications, and Oxford Classification of MEST-C scores. Patients were observed at baseline and at 3, 6, 9, and 12 months and the following indicators were recorded: 24-hour proteinuria, serum creatinine, eGFR, serum albumin, urinary red blood cell (RBC) count, alanine aminotransferase, aspartate aminotransferase, triglyceride, total cholesterol, immunoglobulin A (IgA), immunoglobulin G (IgG), and immunoglobulin M (IgM). Medication prescription data for glucocorticoids, immunosuppressants, renin-angiotensin-aldosterone system inhibitors, and sodium–glucose transport protein 2 inhibitors were recorded. eGFR was calculated using the Chronic Kidney Disease Epidemiology Collaboration equation (9). Kidney biopsies were assessed using the Oxford Classification of MEST-C score (M, mesangial hypercellularity; E, endocapillary hypercellularity; S, segmental glomerulosclerosis; T, tubular atrophy or interstitial fibrosis; and C, crescents) (10).

### 2.3 Outcomes and follow-up

The primary outcomes were percentage changes in 24-hour proteinuria and eGFR during follow-up. Secondary outcomes included changes from baseline in serum creatinine, albumin, urinary RBC count, and proportion of remission during follow-up. Based on clinical treatment outcomes, therapeutic responses were categorized as complete, partial, or no remission. Complete remission (CR) was defined as 24-hour proteinuria ≤ 0.3 g, serum albumin *>* 35 g/L, and stable renal function (a decrease in eGFR ≤ 30%). Partial remission (PR) was defined when 24-hour proteinuria decreased by *>* 50% with stable renal function but did not achieve CR. No remission (NR) was defined as when the above criteria were not met. Exploratory stratified analyses were conducted for baseline proteinuria, baseline eGFR, telitacicept treatment duration, and Oxford Classification. Adverse events during follow-up, including symptoms, signs, diseases, and laboratory test abnormalities, were recorded.

### 2.4 Statistical analyses

Categorical variables are expressed as numbers and percentages and were analyzed using the chi-square test or Fisher’s exact test. Normally distributed continuous variables are expressed as mean ± standard deviation. Paired-sample t-test was adopted for intragroup comparisons, while one-way analysis of variance (one-way ANOVA) was applied for intergroup comparisons. Continuous variables that did not follow a normal distribution were expressed as medians (interquartile range). Wilcoxon signed-rank test was used for intragroup comparisons, while Mann-Whitney U test or Kruskal-Wallis H test was adopted for intergroup comparisons. Statistical significance was set at a two-tailed *P* < 0.05. Descriptive statistics, baseline comparisons, and conventional statistical tests (e.g., t-test, chi-square test, ANOVA, and non-parametric tests) were performed in SPSS 27.0 (IBM Corp., Armonk, NY, USA). Three-group 1:1:1 propensity score matching was accomplished using R version 4.2.3 (R Foundation for Statistical Computing, Vienna, Austria).

## 3 Results

### 3.1 Baseline characteristics

The study recruited 373 patients diagnosed with IgAN as confirmed by renal biopsy; 24 patients treated with telitacicept were included in the analysis according to the inclusion and exclusion criteria. The mean patient age was 42.71years and 32(44.44%) patients were female. The patients had a median disease duration of 5.00 years prior to the study. The baseline proteinuria was 2.57 ± 1.30 g/day, and the baseline eGFR was 44.20 (27.23, 64.03) mL/min/1.73 m^2^. In the telitacicept group and the supportive treatment group, the baseline urinary RBC count was significantly lower than that in the IS group [14.90 (6.75, 108.60) and 21.15 (15.50, 21.15) cells/μL vs 77.15 (33.50, 175.75) cells/μL, *P* = 0.008]. In the telitacicept group, eight patients were treated with telitacicept 240 mg per week subcutaneously, and the remaining patients received 160 mg per week. All patients in the IS treatment group received oral glucocorticoids; among them, 12 received MMF orally. Thirteen patients in the telitacicept group, 12patients in the supportive treatment group, and ten in the IS treatment group received SGLT2i. No significant differences were observed among the three groups in terms of age, sex, disease duration, 24-hour proteinuria, creatinine, eGFR, albumin, IgA, IgG, IgM, triglycerides, and total cholesterol. The baseline clinical characteristics of the patients are presented in **Table 1**.

**Table 1:** Baseline characteristics of patients.

Variable	Telitacicept treatment (n = 24)	Supportive treatment (n = 24)	IS treatment (n = 24)	F/H/χ²	Df	P
Age, years	43.83 ± 10.28	41.21 ± 9.06	43.08 ± 9.65	0.138	2	0.886
Disease duration, years	4.50 (2.25, 9.50)	5.50 (3.00, 8.75)	4.00 (3.00, 9.75)	0.396	2	0.820
Female, n (%)	11 (45.83)	11 (45.83)	10 (41.67)	0.112	2	0.945
BMI, kg/m^2^	23.38 ± 1.83	23.62 ± 2.05	23.65 ± 1.67	0.144	2	0.866
SBP, mmHg	132.75 ± 12.25	134.29 ± 10.70	132.28 ± 14.40	0.243	2	0.785
DBP, mmHg	79.83 ± 9.97	78.37 ± 8.37	79.96 ± 7.41	0.249	2	0.781
Serum albumin, g/L	36.75 (31.50, 39.98)	37.60 (33.60, 39.20)	35.70 (30.92, 40.52)	1.010	2	0.604
Creatinine, μmol/L	156.75 (111.65, 199.60)	148.50 (101.00, 190.80)	151.70 (103.60, 208.70)	0.087	2	0.957
eGFR, mL/min/1.73 m^2^	44.10 (27.15, 60.95)	46.15 (28.02, 67.05)	44.55 (27.70, 63.95)	0.192	2	0.920
Urine RBC, cells/μL	14.90 (6.75, 108.60)	21.15 (15.50, 21.15)	77.15 (33.50, 175.75)	9.587	2	0.008
24-hour proteinuria, g/day	2.54 ± 1.17	2.59 ± 1.54	2.56 ± 1.21	0.008	2	0.992
Triglyceride, mmol/L	1.73 ± 0.73	1.62 ± 0.43	1.69 ± 0.54	0.242	2	0.786
Total cholesterol, mmol/L	5.18 ± 0.92	4.99 ± 0.80	5.03 ± 0.78	0.467	2	0.708
Serum IgA, g/L	2.84 ± 1.09	3.05 ± 1.02	2.67 ± 0.94	0.857	2	0.429
Serum IgG, g/L	9.47 ± 2.63	10.08 ± 1.89	9.97 ± 2.11	0.514	2	0.600
Serum IgM, g/L	1.24 (0.88, 1.64)	1.45 (1.00, 1.68)	1.25 (1.02, 1.70)	0.646	2	0.724
Oxford classification						
M1, n (%)	15 (62.50)	17 (70.83)	17 (70.83)	0.511	2	0.774
E1, n (%)	14 (58.33)	13 (54.17)	15 (62.50)	0.184	2	0.912
S1, n (%)	11 (45.83)	10 (41.67)	11 (45.83)	0.112	2	0.945
T≥1, n (%)	11 (45.83)	13 (54.17)	10 (41.67)	0.780	2	0.677
C≥1, n (%)	10 (41.67)	12 (50.00)	10 (41.67)	0.566	2	0.799

IS, immunosuppressive; BMI, body mass index; SBP, systolic blood pressure; DBP, diastolic blood pressure; eGFR, estimated glomerular filtration rate; RBC, red blood cell; IgA, immunoglobulin A; IgG, immunoglobulin G; IgM, immunoglobulin M; M, mesangial hypercellularity; E, endocapillary hypercellularity; S, segmental glomerulosclerosis; T, tubular atrophy/interstitial fibrosis.

### 3.2 Primary outcomes

**Figure 1:**
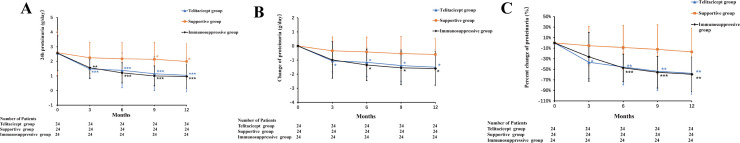
24-hour proteinuria **(A)**, Change of proteinuria **(B)** and percentage change of proteinuria **(C)** during each follow-up period in the three groups. The data are expressed as mean ± standard deviation. **(A)**: baseline 24-hour proteinuria as reference. **(B, C)**: supportive treatment group as reference. *p < 0.05, **p < 0.01, ***p < 0.001.

Changes in proteinuria in each group are shown in **Figure 1** and **Table 2**. At 3 months of treatment, there were significant decreases in 24-hour proteinuria compared to baseline in both the telitacicept group (1.48 ± 0.68 vs 2.54 ± 1.17 g/day, *P* < 0.001) and the IS group (1.56 ± 0.72 vs 2.56 ± 1.21 g/day, *P* = 0.001), and this trend continued until 12 months of follow-up (telitacicept group: 1.04 ± 1.13 vs 2.54 ± 1.17 g/day, *P* < 0.001; IS group: 0.96 ± 0.80 vs 2.56 ± 1.21 g/day, *P* < 0.001). In the supportive treatment group, significant differences in 24-hour proteinuria levels relative to baseline were only detected at month 9 (2.05 ± 1.17 vs 2.59 ± 1.54 g/day, *P* = 0.039) and month 12 (1.99 ± 1.22 vs 2.59 ± 1.54 g/day, *P* = 0.017). Overall, the mean changes in the telitacicept group were superior to those in the supportive treatment group at 3 months (−1.06 ± 0.78 vs −0.34 ± 0.98 g/day, *P* = 0.02), 6 months (−1.16 ± 1.01 vs −0.42 ± 1.05 g/day, *P* = 0.019), 9 months (−1.39 ± 1.14 vs −0.54 ± 1.21 g/day, *P* = 0.046), and 12 months (−1.50 ± 1.23 vs −0.60 ± 1.15 g/day, *P* = 0.034). There was no statistically significant difference in changes in proteinuria between the telitacicept and IS treatment group at any follow-up point (*P* > 0.05). Overall, the mean percentage changes in the telitacicept group from baseline to month 3 (−36.61 ± 31.23% vs −5.24 ± 36.82%, *P* = 0.019), month 6 (−45.55 ± 34.08% vs −8.64 ± 42.07%, *P* = 0.001), month 9 (−53.68 ± 36.24% vs –12.23 ± 46.52%, *P* = 0.001), and month 12 (−57.61 ± 39.96% vs −17.11 ± 45.25%, *P* = 0.002) were significantly decreased compared with the supportive treatment group. The mean percentage change in proteinuria in the IS treatment group from baseline to month 3 showed no statistically significant difference compared with the supportive treatment group (*P* > 0.05), whereas at month 6 (−47.26 ± 26.05% vs −8.64 ± 42.01%, *P* < 0.001), month 9 (−55.65 ± 30.01% vs –12.23 ± 46.52%, *P* < 0.001), and month 12 (−59.49 ± 32.76% vs −17.11 ± 45.25%, *P* = 0.001), statistically significant differences were observed. However, no significant difference was observed in the mean percentage change between the telitacicept and the IS treatment groups during follow-up (*P* > 0.05).

**Table 2:** Change of proteinuria and eGFR in the three groups.

Change of proteinuria (g/day)	Month 3		Month 6		Month 9		Month12	
	F	P	F	P	F	P	F	P
	3.458	0.037	5.142	0.008	5.100	0.09	5.130	0.008
Telitacicept treatment	−1.06 ± 0.78*		−1.16 ± 1.01*		−1.39 ± 1.14*		−1.50 ± 1.23*	
Supportive treatment	−0.34 ± 0.98		−0.42 ± 1.05		−0.54 ± 1.21		−0.60 ± 1.14	
IS treatment	−0.99 ± 1.30*		−1.35 ± 1.11**		−1.55 ± 1.20*		−1.60 ± 1.22*	
Percentage change of proteinuria (%)	F	P	F	P	F	P	F	P
	4.107	0.021	9.496	< 0.001	9.889	< 0.001	8.749	< 0.001
Telitacicept treatment	−36.61 ± 31.23 *		−45.55 ± 34.08 **		−53.68 ± 36.24 **		−57.61 ± 39.94 **	
Supportive treatment	−5.24 ± 36.82		−8.64 ± 42.07		−12.23 ± 46.52		−17.11 ± 45.25	
IS treatment	−26.36 ± 46.43		−47.26 ± 26.05 ***		−55.65 ± 30.01 ***		−59.49 ± 32.76 **	
Change of eGFR (mL/min/1.73 m2)	F	P	F	P	F	P	F	P
	19.568	< 0.001	11.726	0.003	8.925	0.012	8.567	0.014
Telitacicept treatment	−0.05 (0.85, 1.30) **		−0.40 (−2.00, 1.35) **		−1.55 (−4.00, 1.25) *		−2.10 (−5.75, 0.70) *	
Supportive treatment	−2.35 (−5.80, −0.75)		−3.60 (−9.00, −1.50)		−4.95 (−8.95, −1.85)		−6.05 (−9.55, −2.20)	
IS treatment	0.30 (−0.80, 1.20) ***		−0.85 (−1.95, 0.05) *		−1.60 (−3.10, −0.25) *		−2.65 (−4.35, −0.75) *	
Percentage change of eGFR (%)	F	P	F	P	F	P	F	P
	20.304	< 0.001	13.715	0.001	13.556	0.001	11.073	0.004
Telitacicept treatment	−0.06 (−2.07, 4.06) **		−0.64 (−5.49, 4.58) **		−3.97 (−8.99, 5.60) *		−4.23 (−11.26, 2.17) *	
Supportive treatment	−6.59 (−9.72, −3.23)		−9.17 (−12.55, −4.15)		−11.44 (−14.20, −5.34)		−13.87 (−18.00, −6.30)	
IS treatment	0.41 (−1.52, 2.67) ***		−1.90 (−4.28, 0.39) **		−3.56 (−5.79, −0.99) **		−5.65 (−6.77, −2.37) **	

IS, immunosuppressive; eGFR, estimated glomerular filtration rate. The supportive treatment group was used as the reference. *P < 0.05, **P < 0.01, ***P < 0.001.

The changes in eGFR and percentage changes in eGFR for each group are presented in **Table 2**. After 12 months follow-up, the telitacicept group exhibited a minor decline in eGFR of 2.1mL/min/1.73 m2 (−4.23%), while the supportive treatment group and IS treatment group showed declines of 6.05 mL/min/1.73 m^2^ (−13.87%) and 2.65 mL/min/1.73 m2 (−5.65%), respectively (telitacicept group vs supportive treatment group, *P* = 0.021, telitacicept group vs IS treatment group, P > 0.05). Concurrently, the differences among the three groups exhibited similar trends at 3, 6, and 9 months.

### 3.3 Secondary outcomes

As shown in **Table 3**, during follow-up, serum albumin levels in the telitacicept group showed significant increases compared with baseline (*P <*0.001*)*. In the supportive treatment group, albumin levels increased from baseline only at 6, 9 and 12 months. During follow-up, serum creatinine levels in the telitacicept group remained stable compared with baseline, whereas the supportive treatment group showed a significant increase compared with baseline (*P < 0.001*). In all three groups, urine RBC counts decreased significantly from baseline at 9 months, which persisted until the 12-month follow-up.

**Table 3:** Secondary outcomes in the three groups.

	Month 3	Z	Month 6	Z	Month 9	Z	Month 12	Z
Serum albumin (g/L)								
Telitacicept treatment	41.50 (38.30, 43.28) ***	4.115	40.95 (39.50, 43.85) ***	4.143	41.45 (39.75, 43.30) ***	4.143	41.35 (39.55, 43.75) ***	3.657
Supportive treatment	38.60 (35.45, 40.20)	1.645	39.00 (36.50, 40.15)*	1.875	39.20 (36.60, 40.65) **	2.576	40.35 (36.30, 42.15) **	2.593
IS treatment	38.30 (33.10, 41.25) ***	4.198	39.95 (35.40, 41.00) ***	3.863	40.25 (37.25, 42.40) ***	4.086	41.70 (39.20, 42.95) ***	4.153
Creatinine(μmol/L)								
Telitacicept treatment	161.00 (117.50, 198.75)	0.198	166.05 (120.15, 202.40)	0.882	167.95 (119.90, 200.60)	1.229	168.85 (123.50, 202.75)	1.883
Supportive treatment	156.25 (116.25, 196.10) ***	3.972	160.65 (117.60, 195.40) ***	3.686	167.05 (121.15, 196.95) ***	3.972	177.15 (125.15, 199.50) ***	3.829
IS treatment	155.50 (105.60, 206.50)	0.414	154.55 (105.60, 212.50)	1.372	155.90 (110.25, 213.95)	1.571	157.00 (112.30, 216.25)*	2.143
Urine RBC, cells/μL								
Telitacicept treatment	11.75 (4.65, 29.05)***	−3.429	9.10 (4.75, 28.45) ***	−3.772	8.45 (2.40, 23.80) **	−3.114	7.00 (5.10, 10.4) **	−3.068
Supportive treatment	22.50 (10.20, 77.25)*	−2.229	19.60 (8.90, 104.00)	−1.315	20.30 (9.85, 77.80)*	−2.403	15.50 (10.20, 70.25) ***	−3.357
IS treatment	71.30 (37.95, 123.00)**	−2.686	52.00 (23.60, 101.90)*	−2.543	39.65 (13.95, 105.20)*	−2.20	34.00 (15.25, 106.60)*	−2.372

IS, immunosuppressive; eGFR, estimated glomerular filtration rate. RBC, red blood cell. Baseline as reference. *P < 0.05, **P < 0.01, ***P < 0.001.

### 3.4 Response time and rate

The remission rates are shown in **Table 4**. The telitacicept and IS treatment groups achieved significantly better remission than the supportive treatment group. The remission rates (CR+PR) were similar between the telitacicept group and immunosuppressive treatment group at 3–12 months (*P* > 0.05). At 6 months, the overall response rates were 66.67% and 54.17% (*P* = 0.376). After 12 months, the remission rates were 79.16% in the telitacicept group and 83.33% in the IS treatment group. However, the CR rate in the telitacicept group (37.50%) was higher than that in the immunosuppressive treatment group (25.00%), although this difference was not statistically significant (*P* = 0.350).

**Table 4:** Remission rates at 3, 6, 9, and 12 months after treatment.

Variables	Telitacicept treatment (n = 24)	Supportive treatment (n = 24)	IS treatment (n = 24)	χ²	df	P
3 months, n (%)				2.215	2	0.330
NR	16 (66.67)	20 (83.33)	16 (66.67)			
PR	8 (33.33)	4 (16.67)	8(33.3)			
CR	0 (0)	0 (0)	0 (0)			
6 months, n (%)				10.811	2	0.004
NR	8 (33.33)	19 (79.16)	11 (45.83)			
PR	12 (50.00)	4 (16.67)	9 (37.50)			
CR	4 (16.67)	1 (4.17)	4 (16.67)			
9 months, n (%)				8.136	2	0.017
NR	8 (33.33)	17 (70.83)	9 (37.50)			
PR	12 (50.00)	6 (25.00)	10 (41.67)			
CR	4 (16.67)	1 (4.17)	5 (20.83)			
12 months, n (%)				13.875	2	*<*0.001
NR	5 (20.83)	15 (62.50)	4 (16.67)			
PR	10 (41.67)	7 (29.17)	14(58.33)			
CR	9 (37.50)	2 (8.33)	6 (25.00)			

IS, immunosuppressive; NR, no remission; PR, partial remission; CR, complete remission.

### 3.5 Exploratory stratified analysis

**Figure 2:**
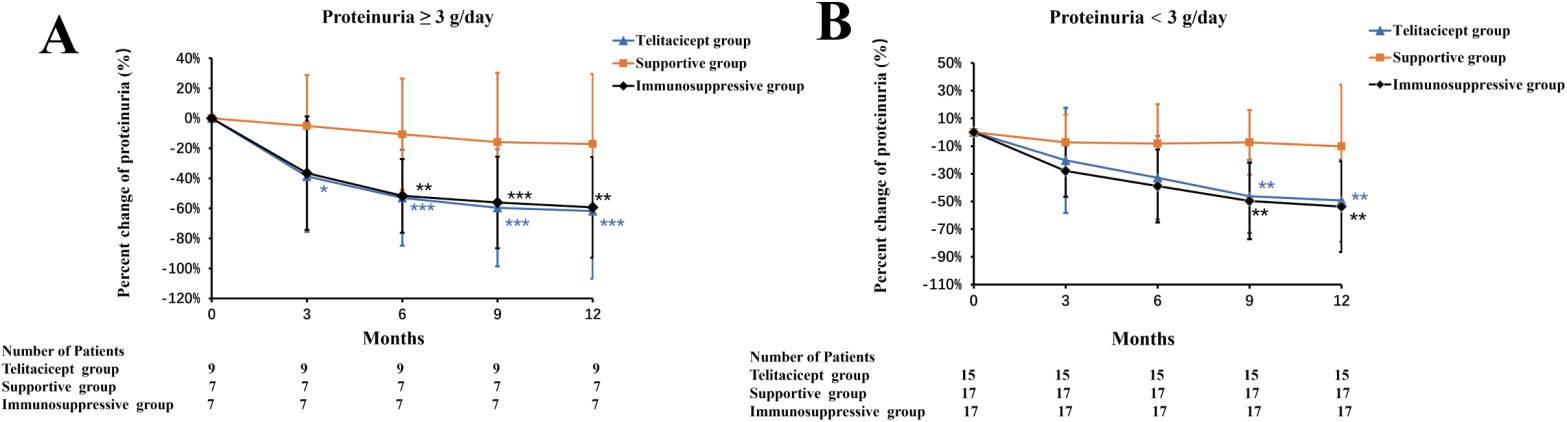
Percentage change of proteinuria in the patients with proteinuria ≥ 3 g **(A)** and proteinuria < 3 g **(B)**. The data are expressed as mean ± standard deviation. Supportive treatment group as reference. *p < 0.05, **p < 0.01, ***p < 0.001.

We conducted exploratory stratified analysis based on baseline 24-hour proteinuria. The percentage changes in proteinuria were compared among the different groups (**Figure 2**). In patients with proteinuria ≥3 g/day, the telitacicept group demonstrated significantly superior proteinuria reduction rates compared with the supportive treatment group at month 3 (−38.64 ± 37.05% vs.−5.00 ± 33.84%, *P* = 0.023), month 6 (−52.93 ± 32.02% vs.−10.55 ± 37.07%, *P* < 0.001), month 9 (−59.64 ± 38.99%vs.−15.81± 46.17%, *P < 0.001*), and month 12 (−61.79 ± 44.99% vs. −17.07± 46.63%, *P* < 0.001). For patients with proteinuria < 3 g/day, the telitacicept group was superior to the supportive treatment group only at 9 months (−46.32 ± 26.39% vs. −7.32 ± 26.39%, *P* = 0.002) and 12 months (−49.31 ± 29.63% vs. −10.32 ± 44.60%, *P* = 0.004). However, regardless of the baseline 24-hour proteinuria, no significant difference in the percentage change in proteinuria was observed between the telitacicept group and IS treatment group at these points (*P* > 0.05).

**Figure 3:**
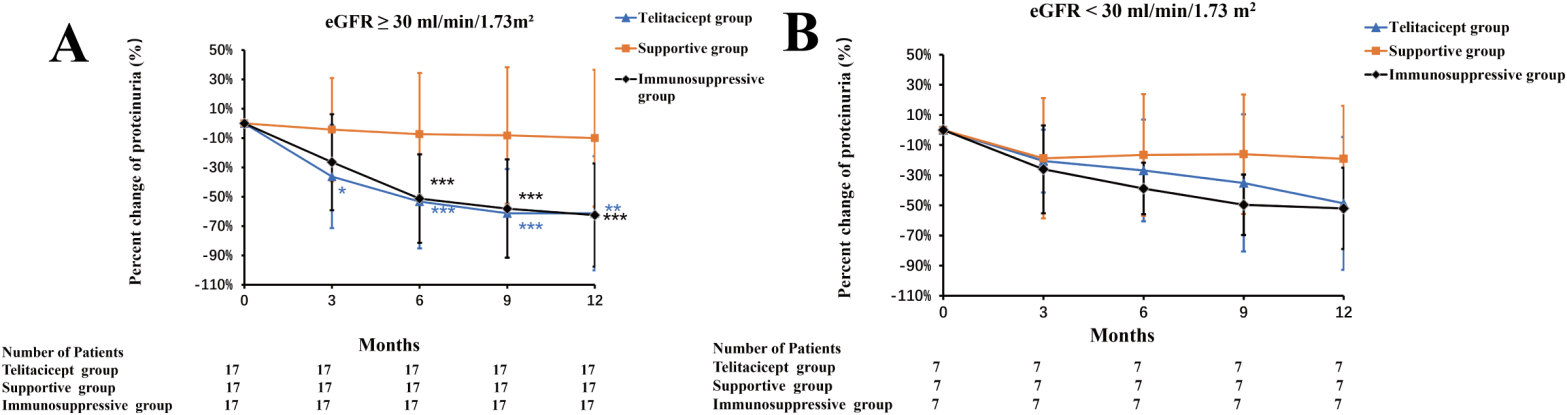
Percentage change of proteinuria in the patients with eGFR ≥ 30 ml/min/1.73 m^2^ **(A)** and eGFR < 30 ml/min/1.73 m^2^ **(B)**. The data are expressed as mean ± standard deviation. Supportive treatment group as reference. *p < 0.05, **p < 0.01.

We performed exploratory stratified analysis based on baseline eGFR. For patients with eGFR ≥ 30 ml/min/1.73 m^2^, the percentage change in proteinuria in the telitacicept group was significantly better than that in the supportive treatment group at month 3 (−36.34 ± 35.21% vs. −4.27 ± 35.21%, *P* = 0.042), month 6 (−53.27 ± 32.00% vs.−7.3 ± 41.65%, *P <* 0.001), month 9 (−61.32 ± 30.17% vs.−8.23 ± 46.56%, *P <* 0.001), and month 12 (−61.27 ± 38.96% vs.−10.04 ± 46.66%, *P* = 0.001). However, there was no significant difference in the percentage change in proteinuria between the telitacicept group and IS treatment group during follow-up (*P* > 0.05). For patients with eGFR < 30 ml/min/1.73 m^2^, there was no significant difference in the percentage change of proteinuria among the three groups (*P* > 0.05) (**Figure 3**).

**Figure 4:**
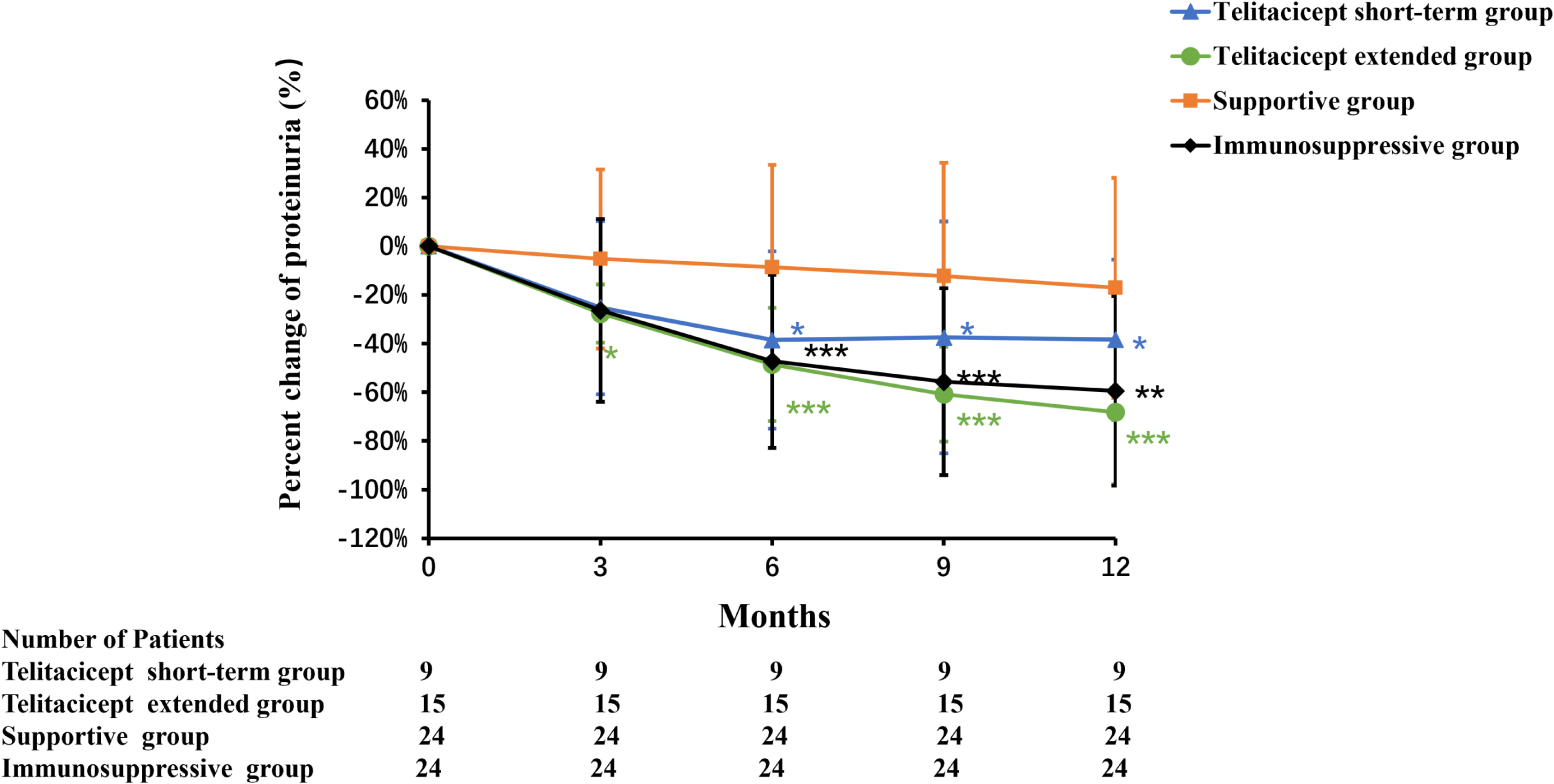
Percentage change of proteinuria in the patients with telitacicept short-term treatment and the extended group. The data are expressed as mean ± standard deviation. Supportive treatment group as reference. *p < 0.05, **p < 0.01, ***p < 0.001.

Exploratory stratified analysis based on the duration of telitacicept treatment defined the short-term treatment group as ≤ 6 months and the extended treatment group as > 6 months. We found that at 12 months, the percentage changes of proteinuria were −38.37 ± 38.92% and −65.16 ± 29.62% in the short-term and extended treatment group, respectively, both significantly superior to the supportive treatment group (−17.15 ± 45.25%, *P* < 0.05). During follow-up, starting from 6 months, the percentage changes in proteinuria in the extended treatment group consistently showed significant improvement compared with the short-term treatment group (*P* < 0.05) (**Figure 4**).

**Figure 5:**
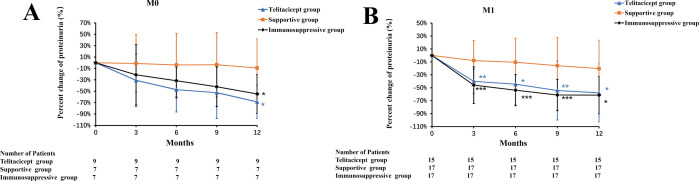
Percentage change of proteinuria in the patients with Oxford Classification of M0 **(A)** and M1 score **(B)**. The data are expressed as mean ± standard deviation. Supportive treatment group as reference. *p < 0.05, **p < 0.01, ***p < 0.001.

**Figure 6:**
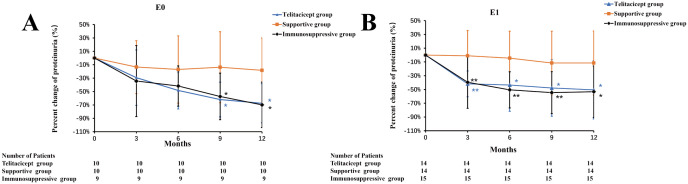
Percentage change of proteinuria in the patients with Oxford Classification of E0 **(A)** and E1 score **(B)**. The data are expressed as mean ± standard deviation. Supportive treatment group as reference. *p < 0.05, **p < 0.01, ***p < 0.001.

**Figure 7:**
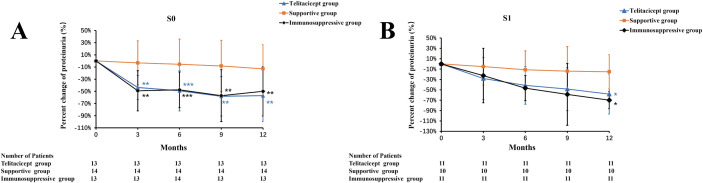
Percentage change of proteinuria in the patients with Oxford Classification of S0 **(A)** and S1 score **(B)**. The data are expressed as mean ± standard deviation. Supportive treatment group as reference. *p < 0.05, **p < 0.01, ***p < 0.001.

**Figure 8:**
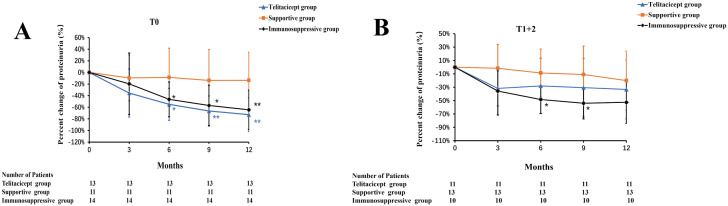
Percentage change of proteinuria in the patients with Oxford Classification of T0 **(A)** and T1+2 score **(B)**. The data are expressed as mean ± standard deviation. Supportive treatment group as reference. *p < 0.05, **p < 0.01, ***p < 0.001.

**Figure 9:**
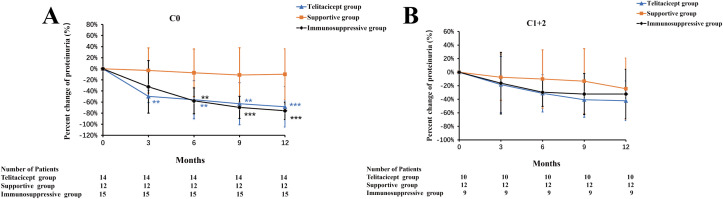
Percentage change of proteinuria in the patients with Oxford Classification of C0 **(A)** and C1+2 score **(B)**. The data are expressed as mean ± standard deviation. Supportive treatment group as reference. *p < 0.05, **p < 0.01, ***p < 0.001.

The percentage changes of proteinuria in exploratory stratified analysis according to Oxford Classification of MEST-C score (M0 vs M1, E0 vs E1, S0 vs S1, T0 vs T1 and T2, C0 vs C1 and C2) were shown in **Figures 5**–**9**. Interestingly, proteinuria decreased significantly in the telitacicept and IS group versus the supportive treatment group in all subgroups (M0 vs M1, E0 vs E1, S0 vs S1) at Month 12. We found that the difference was not significant in three groups with more severe pathological changes (T1/T2 and C1/C2).

### 3.6 Adverse events

The details of the adverse events of the three groups are presented in **Table 5**. No serious adverse events were observed during treatment. The overall incidence of adverse events was higher in the IS treatment group (*P* = 0.011). The IS treatment group exhibited a higher incidence of respiratory infections, gastrointestinal discomfort, and fatigue. Conversely, injection site pain, redness, and swelling were more common in patients treated with telitacicept, which were related to subcutaneous administration. Overall, telitacicept demonstrated a more favorable safety profile with fewer adverse events than IS treatment (*P* = 0.009).

**Table 5:** Adverse events in the three groups.

Events, *n* (%)	Telitacicept treatment (n = 24)	Supportive treatment (n = 24)	IS treatment (n = 24)	χ²	df	P
Any adverse events	8 (33.33)	8 (33.33)	17 (70.83)	9.489	2	0.009
SAEs	0 (0)	0 (0)	0 (0)			
Injection site reactions	4 (16.67)	0 (0)	0 (0)			
Respiratory tract infection	2 (8.33)	1 (4.17)	6 (25.00)			
Urinary tract infection	0 (0)	1 (4.17)	2 (8.33)			
Gastrointestinal symptoms	0 (0)	0 (0)	3 (12.50)			
Fatigue	0 (0)	0 (0)	2 (8.33)			
Abnormal liver function	2 (8.33)	0 (0)	2 (8.33)			
Insomnia	0 (0)	0 (0)	1 (4.17)			
Giddy	0 (0)	2 (8.33)	2 (8.33)			
Hyperkalemia	1 (4.16)	2 (8.33)	0 (0)			
Dry cough	0 (0)	2 (8.33)	0 (0)			

IS, immunosuppressive; SAE, serious adverse event.

## 4 Discussion

Although IgAN was first described over 50 years ago and significant advances have been made in its pathophysiology to date, the appropriate treatment, especially IS therapy for IgAN, remains uncertain (11). The strongest prognostic factor for IgAN is proteinuria, with effects that are dose-dependent and independent of other risk factors (12). While previous guidelines recommended controlling proteinuria to < 0.5 g/day as a treatment target for patients with IgAN, a recent cohort study of 1,530 IgAN patients found that those with proteinuria levels < 0.3 g/day had a lower risk of kidney failure (13).

Telitacicept is a novel dual-target recombinant fusion protein that is a product of the extracellular segment of the BAFF/APRIL soluble receptor and the IgG1 Fc segment. It can simultaneously neutralize BAFF and APRIL, inhibit the development and maturation of B cells and their differentiation into plasma cells, thereby affecting the production of autoantibodies by abnormal plasma cells (14, 15). The results of a phase II clinical trial of telitacicept in patients with IgAN showed that after 24 weeks of treatment with 160 or 240 mg telitacicept per week, proteinuria levels decreased by 25% and 49%, respectively, compared with baseline values. Furthermore, serum IgA, IgG, and IgM levels significantly decreased, while eGFR remained stable during the treatment period (16). Zan et al. have also reported that telitacicept reduces proteinuria by decreasing IgA-containing immune complexes and circulating Gd-IgA1 (17).

In the past two years, some retrospective clinical studies on the treatment of IgAN with telitacicept have also been published (**Table 6**). A portion of relevant studies conducted intragroup comparative analysis based on clinical indicators prior to and following telitacicept intervention. The remaining studies made comparisons between telitacicept and different immune intervention, as well as evaluated the clinical value of combined medication regimens. Multiple studies in China have reported that the use of MMF alone or in combination with glucocorticoids is beneficial in patients with progressive IgAN (18, 19). The comparison of the efficacy of immunosuppressants and telitacicept in treating IgAN remains a hotspot in current research, and conclusions from different studies remain inconsistent. Dong et al. found that telitacicept demonstrated similar clinical efficacy to traditional IS therapy at 3 months in reducing proteinuria, with fewer adverse events observed in the telitacicept group, preserved eGFR levels were observed in the telitacicept group, whereas eGFR levels decreased in the other two groups (20). Consistent with the above-mentioned studies, the present study adopted propensity score matching to compare the efficacy of telitacicept with the supportive group and IS group. In contrast, our study had a maximum follow-up of 12 months, much longer than the 3-month follow-up in prior studies. We believe that a continuous 12-month follow-up is critically important for evaluating the therapeutic efficacy and long-term renal prognosis of telitacicept. Moreover, the patients enrolled in our study had higher baseline 24-hour proteinuria and lower baseline eGFR levels, indicating more severe baseline renal impairment. The therapeutic response to telitacicept in this population with more severe renal damage also warrants further in-depth investigation. Given the significant regional heterogeneity in the prevalence of IgAN worldwide, this study was conducted among patients from East China, where the incidence of IgAN is significantly higher than that in West China as reported in the above literature. Our findings contribute to identifying the efficacy differences of telitacicept in populations from different geographic regions. Additionally, we performed exploratory analyses on the associations of baseline proteinuria, baseline eGFR, treatment duration, and different pathological subtypes with therapeutic response in this study. He et al. found that after 12 months of treatment, telitacicept combined with low-dose steroids performed better in reducing proteinuria (reduction of −62.5% compared with −52.9% in the MMF combined with low-dose steroids group, *P* = 0.041) and stabilizing renal function (eGFR improved by 4.1%, while it decreased by 5.3% in the MMF combined with low-dose steroids group, *P* = 0.085) (21). Zeng et al. found that the telitacicept + glucocorticoid/MMF group showed a significantly greater reduction in proteinuria (-1.32 g/day versus -0.94 g/day) and exhibited better eGFR slope (2.74 mL/min/1.73 m2/year versus -0.56 mL/min/1.73 m^2^/year) compared to the telitacicept group (22), but experienced a greater overall rate of non-serious side effects compared to the telitacicept group (28.2% versus 11.2%).Our results indicated that patients receiving immune intervention experienced a higher rate of adverse events than those treated with telitacicept or supportive therapy. Infection constituted the major adverse event. Clinically, attention should be paid to potential infectious complications during combined IS therapy with telitacicept, and prophylactic infection prevention measures are recommended as appropriate.

**Table 6:** Retrospective study on the treatment of IgAN with telitacicept.

Reference	Experimental design and follow-up time	Group	Conclusion
Luo et al. (26)	Single-center, retrospective 24 weeks	Telitacicept 160 mg (n=11)	24h proteinuria decreased from a baseline of 1.77g to 1.04g at week 24.
Shu et al. (27)	Single-center, retrospective 24 months	Telitacicept plus low-dose MMF(n=24)	There were noteworthy reductions in proteinuria at 3, 6, 9, 12, 15, 18, 21 and 24 months when compared to the baseline levels. All patients maintained stable eGFR during follow-up times.
Weng et al. (23)	Single-center, retrospective 12 months	Telitacicept treatment (n=41, Extended group, n = 26, Short-term group, n = 15) ACEI/ARB group (n = 41)	The percent change in proteinuria from baseline of extended group, short-term group and ACEI/ARB group were –56.8 ± 23.5% (P < 0.01), –28.6 ± 65.6% (P = 0.09) and –0.3 ± 57.0% at month 12. eGFR decline in telitacicept groups were slower compared with the ACEI/ARB group.
Dong et al. (20)	Single-center, retrospective 3 months	Telitacicept (n = 21) Supportive treatment (n = 21) IS treatment (n = 21)	Telitacicept reduced proteinuria by 0.72g/d (54.6%) from baseline, compared with a reduction of 0.18 g/d (20%) in the supportive treatment group and 1.12 g/d (72.1%) in the IS treatment group. Preserved eGFR levels were observed in the telitacicept group, whereas eGFR levels decreased in the other two groups.
He et al. (21)	Retrospective, multicenter cohort study 12 months	MMF combined with low-dose steroids (n = 56) Telitacicept combined with low-dose steroids (n = 48)	At 12 months, telitacicept plus low-dose steroids demonstrated superior proteinuria reduction (−62.5% versus −52.9%) and stabilized renal function (4.1% improvement in eGFR rate versus 5.3% decline with MMF, P = 0.085).
Tao et al. (28)	Single-center, retrospective 24 weeks	Telitacicept monotherapy (n = 8) Telitacicept combined with low-dose corticosteroids (n = 8) Telitacicept combined with full-dose corticosteroids or corticosteroids plus other IS (n = 8)	After 12 weeks and 24 weeks of treatment, patients in all three groups showed a significant decrease in 24h proteinuria from baseline. Mean serum creatinine and eGFR levels remained stable in all three groups.
Wang et al. (29)	Single-center, retrospective 24 weeks	Whole telitacicept group (n = 42, newly treated telitacicept, n = 20) Conventional IS group (n = 28)	Telitacicept reduced in proteinuria and increases in eGFR similar to conventional IS therapy. After week 24, the incidence of adverse events was lower for telitacicept than for conventional IS therapy.
Zeng et al. (22)	Retrospective, multicenter cohort study 9 months	Telitacicept monotherapy(n=125) Telitacicept plus GM therapy(n=131)	At 9 months, the telitacicept + GM group showed a significantly greater reduction in proteinuria (−1.32 g/day versus −0.94 g/day) and exhibited better eGFR slope (2.74 mL/min/1.73 m2/year versus −0.56 mL/min/1.73 m2/year) compared to the telitacicept group.

eGFR, estimated glomerular filtration rate; GM, glucocorticoid/mycophenolate mofetil; UACR, urinary albumin-creatinine ratio; IS, immunosuppressants; Scr, serum creatinine; IgAN, immunoglobulin A nephropathy.

Our study demonstrated that telitacicept reduced 24-hour proteinuria by 57.61% from baseline. After 12 months of follow-up, there was no significant difference in the change in eGFR between the telitacicept and IS treatment groups; however, both were significantly better than those in the supportive treatment group. The incidence of adverse events was lower in the telitacicept group than that in the IS treatment group. Furthermore, the telitacicept group also showed high remission rates at each follow-up. We not only analyzed the changes in relevant indicators before and after telitacicept treatment, but also optimized the research design by adopting propensity score matching (balance baseline covariates and reduce confounding bias across three groups). Dual control groups were established, including conventional supportive treatment and IS regimens (glucocorticoids combined with or without MMF), so as to comprehensively and objectively evaluate the clinical efficacy of telitacicept in the treatment of IgAN. Most existing retrospective clinical studies on telitacicept have relatively short observation cycles, generally 3 or 6 months. In contrast, this study extended the follow-up period to 12 months, with outcome indicators assessed every 3 months. This design enables systematic evaluation of symptom improvement trends, long-term therapeutic effects and adverse reaction profiles throughout the whole treatment course. Furthermore, stratified analyses were performed based on core clinical parameters, namely baseline 24-hour proteinuria, eGFR, treatment course and pathological classification. We further explored the optimal population suitable for telitacicept therapy, aiming to provide evidence for individualized clinical medication strategies.

Overall, the remission rate gradually increased with treatment duration, reaching a peak at 12 months. Extending the treatment duration from 6 months to 9–12 months further enhances the ability to reduce proteinuria (23). We found that starting from 6 months, the percentage change in proteinuria in the telitacicept-extended treatment group consistently showed significant improvement compared with the short-term treatment group. This suggests that the application time can be appropriately extended in clinical practice.

The exploratory study found that at 12 months, compared with the supportive treatment group, both the telitacicept and IS groups showed a significant reduction in proteinuria across all groups (M0 vs M1, E0 vs E1, S0 vs S1). However, no significant between-group differences in proteinuria were observed among patients with more severe T and C lesions. Patients with severe T lesions and active C lesions tend to have poor renal prognosis. This pathological feature may explain why telitacicept and IS therapies yield limited efficacy in such populations. The findings provide important implications for individualized treatment strategies in clinical practice. But it is also possible that the time interval between renal biopsy and treatment initiation was relatively long. Hence, baseline pathological findings may not accurately reflect the actual renal pathological changes during treatment. Further studies are therefore required to clarify the therapeutic responses to telitacicept and IS agents among IgAN patients with different pathological characteristics.

The application of telitacicept in the treatment of autoimmune diseases shows promising efficacy, with good tolerability and high safety (24, 25). In a phase II clinical study of telitacicept for the treatment of IgAN, eGFR remained stable during the medication period, and the incidence of adverse events was similar across all groups, all of which were mild to moderate (16). In our study, no serious adverse events occurred in any patient during follow-up. The incidence of adverse events at the telitacicept injection site was relatively high, yet all were mild. In contrast, common adverse events in the IS treatment group included infections and gastrointestinal symptoms. The incidence of adverse events was significantly lower in the telitacicept group than in the IS treatment group.

This study has some limitations. Firstly, this study was a retrospective single-center study with only 24 patients included in each group, resulting in a small sample size and limited level of evidence. According to the *post hoc* power analysis, at a significance level of α=0.05, the sample size of this study could only detect between-group differences with moderate or larger effect sizes (Cohen’s d ≈ 0.8), and there may be a risk of type II errors for differences with smaller effect sizes. Furthermore, the single-center retrospective design may affect the generalizability of the results. Existing studies are mostly small-sample retrospective single-center analyses. Larger sample sizes, multicenter designs and higher-quality clinical researches are required for further verification. In addition, these exploratory analyses were exploratory and hypothesis-generating. Because the sample size was small and no interaction tests were done, the results should be interpreted with caution. Moreover, in this study, 8 patients who received telitacicept were excluded due to incomplete data. Baseline characteristics of excluded and enrolled patients were compared, and no significant differences were observed in age (*P* = 0.802), gender (*P* = 0.681), baseline 24-hour proteinuria (*P* = 0.648), serum creatinine (*P* = 0.749), or eGFR (*P* = 0.717) between the two groups. Although no significant differences were observed in baseline indicators between enrolled and excluded subjects, selection bias still existed in this retrospective analysis. Seriously ill patients were prone to missing follow-up data, and the exclusion of these cases might inevitably cause selection bias and reduce the overall sample size. Last but not least, monitoring indicators were lacking. B-lymphocyte counts were not regularly monitored, making effective comparisons impossible. Our study lacked research on serum Gd-IgA1 levels; therefore, the association between serum Gd-IgA1 levels and proteinuria could not be further evaluated. To further evaluate the efficacy and safety of telitacicept in patients with IgAN, it is necessary to expand the sample size and improve the detection of the relevant indicators. In the future, we look forward to more large-scale clinical trials and real-world studies to further validate the efficacy and safety of telitacicept in treating patients with IgAN, and hope that telitacicept can provide more clinical benefits to these patients.

## Data Availability

The original contributions presented in the study are included in the article/**Supplementary Material**. Further inquiries can be directed to the corresponding authors.
