# Supplementary material for: Real-world study of telitacicept in the treatment of IgA nephropathy
Source: Front Immunol. 2026 May 29;17:1810706. doi: 10.3389/fimmu.2026.1810706 (PMC13260585; doi:10.3389/fimmu.2026.1810706)
Supplement: Supplementary file 1 [file Table1.docx]

Supplementary Table 1. Comparison of baseline covariate characteristics among the first three groups of matches

| Variable | Group1 | Group2 | Group3 | P | \|SMD \| Maximum | Balance |
| --- | --- | --- | --- | --- | --- | --- |
| Age, years | 43.83 ± 10.28 | 48.83 ± 8.28 | 40.35 ± 9.28 | 0.077 | 0.311 | imbalance |
| Female, n (%) | 11 (45.83) | 87 (50.00) | 83 (47.42) | 0.717 | 0.133 | acceptable |
| 24-hour proteinuria, g/day | 2.54 ± 1.17 | 2.04 ± 1.59 | 1.79 ± 1.29 | 0.012 | 0.587 | imbalance |
| Creatinine, μmol/L | 156.75 (111.65, 199.60) | 96.80 (71.47, 122.43) | 95.20 (72.08, 130.97) | <0.001 | 0.839 | imbalance |
| eGFR, mL/min/1.73 m^2^ | 44.10 (27.15, 60.95) | 78.55 (55.15, 102.72) | 76.85 (53.10, 101.70) | <0.001 | 1.116 | imbalance |

Note. Group 1: Telitacicept treatment group; Group 2: Supportive treatment group; Group 3: Immunosuppressive treatment group. eGFR, estimated glomerular filtration rate; SMD, standardized mean difference.

Supplementary Table 2. Preference Score Matching Parameter Settings

| Parameter | setting |
| --- | --- |
| Match covariates | age, gender, 24-hour urine protein quantification, serum creatinine, eGFR |
| Propensity rating model | multinomial logistic regression |
| Matching ratio | 1∶1∶1 |
| Matching algorithm | Hungarian linear assignment |
| Distance metric | Mahalanobis distance (based on 5 standardized covariates) |
| Caliper | 0.2 × SD [logit P (Group1)] = 0.2391 |
| Return to sampling | N0 |
| Random seed | 2026 |
| Original sample size | 24 / 174 / 175 |
| Number of successfully matched triples | 24 |

eGFR, estimated glomerular filtration rate; SD, standard deviation.

Supplementary Table 3. Comparison of covariate characteristics among three baseline groups after matching.

| Variable | Group1 | Group2 | Group3 | P | \|SMD \| Maximum | Balance |
| --- | --- | --- | --- | --- | --- | --- |
| Age, years | 43.83 ± 10.28 | 41.21± 9.06 | 43.08 ± 9.65 | 0.886 | 0.200 | acceptable |
| Female, n (%) | 11 (45.83) | 11 (45.83) | 10 (41.67) | 0.945 | 0.084 | ideal |
| 24-hour proteinuria, g/day | 2.54 ± 1.17 | 2.59 ± 1.54 | 2.56 ± 1.21 | 0.992 | 0.053 | ideal |
| Creatinine, μmol/L | 156.75 (111.65, 199.60) | 148.50 (101.00, 190.80) | 151.70 (103.60, 208.70) | 0.957 | 0.135 | acceptable |
| eGFR, mL/min/1.73 m^2^ | 44.10 (27.15, 60.95) | 46.15 (28.02, 67.05) | 44.55 (27.70, 63.95) | 0.920 | 0.053 | ideal |

Note. Group 1: Telitacicept treatment group; Group 2: Supportive treatment group; Group 3: Immunosuppressive treatment group. eGFR, estimated glomerular filtration rate; SMD, standardized mean difference.

Supplementary Table 4. Comparison of SMD before and after main covariate matching for each pair.

| Variable | Match pre SMD | | | Matched SMD | | |  |
| --- | --- | --- | --- | --- | --- | --- | --- |
|  | Group 1 vs Group 2 | Group 1 vs Group 3 | Group 2 vs Group 3 | Group 1 vs Group 2 | Group 1 vs Group 3 | Group 2 vs Group 3 | |
| Age, years | +0.311 | +0.194 | -0.113 | +0.184 | -0.004 | -0.200 | |
| Female, n (%) | +0.083 | +0.133 | +0.050 | +0.000 | -0.084 | -0.084 | |
| 24-hour proteinuria, g/day | +0.342 | +0.587 | +0.171 | -0.053 | -0.038 | +0.020 | |
| Creatinine, μmol/L | +0.839 | +0.806 | -0.014 | +0.086 | +0.135 | +0.043 | |
| eGFR, mL/min/1.73 m^2^ | -1.116 | -1.071 | +0.033 | -0.053 | -0.031 | +0.023 | |

Note. Group 1: Telitacicept treatment group; Group 2: Supportive treatment group; Group 3: Immunosuppressive treatment group. eGFR, estimated glomerular filtration rate; SMD, standardized mean difference.
